# CoreValve vs. Sapien 3 Transcatheter Aortic Valve Replacement: A Finite Element Analysis Study

**DOI:** 10.3390/bioengineering8050052

**Published:** 2021-04-27

**Authors:** Francesco Nappi, Laura Mazzocchi, Cristiano Spadaccio, David Attias, Irina Timofeva, Laurent Macron, Adelaide Iervolino, Simone Morganti, Ferdinando Auricchio

**Affiliations:** 1 Department of Cardiac Surgery, Centre Cardiologique du Nord, 93200 Saint Denis, France; 2 Department of Civil Engineering and Architecture, University of Pavia, 27100 Pavia, Italy; laura.mazzocchi02@universitadipavia.it (L.M.); auricchio@unipv.it (F.A.); 3 Institute of Cardiovascular and Medical Sciences, University of Glasgow, Glasgow G12 8QQ, UK; cristianospadaccio@gmail.com; 4 Department of Cardiology, Centre Cardiologique du Nord, 93200 Saint Denis, France; d.attias@ccncardio.com; 5 Department of Imaging, Centre Cardiologique du Nord, 93200 Saint Denis, France; dr.irina.timofeeva@gmail.com (I.T.); laurentmacron@gmail.com (L.M.); 6 Department of Cardiovascular Sciences, Fondazione Policlinico Universitario A. Gemelli IRCSS, 00168 Rome, Italy; adelaide.iervolino@libero.it; 7 Department of Electrical, Computer, and Biomedical Engineering, University of Pavia, 27100 Pavia, Italy; simone.morganti@unipv.it

**Keywords:** cardiac tissue-engineered valves, transcatheter aortic valve replacement, transcatheter heart valve thrombosis, computerized angiography tomography, finite element analysis

## Abstract

Aim: to investigate the factors implied in the development of postoperative complications in both self-expandable and balloon-expandable transcatheter heart valves by means of finite element analysis (FEA). Materials and methods: FEA was integrated into CT scans to investigate two cases of postoperative device failure for valve thrombosis after the successful implantation of a CoreValve and a Sapien 3 valve. Data were then compared with two patients who had undergone uncomplicated transcatheter heart valve replacement (TAVR) with the same types of valves. Results: Computational biomechanical modeling showed calcifications persisting after device expansion, not visible on the CT scan. These calcifications determined geometrical distortion and elliptical deformation of the valve predisposing to hemodynamic disturbances and potential thrombosis. Increased regional stress was also identified in correspondence to the areas of distortion with the associated paravalvular leak. Conclusion: the use of FEA as an adjunct to preoperative imaging might assist patient selection and procedure planning as well as help in the detection and prevention of TAVR complications.

## 1. Introduction

Transcatheter aortic valve replacement (TAVR) has been a standard of care for a growing number of patients with severe aortic valve stenosis, and its efficacy and safety is supported by large randomized studies [1,2,3,4,5,6]. However, several device-related and procedure-related complications still hamper the clinical outcomes of TAVR. Among those, clinical and subclinical valve thrombosis [7,8], paravalvular regurgitation [9], incomplete expansion of TAVR devices [10], reduced leaflet motion (RLM) and hypo-attenuated leaflet thickening (HALT) represent major concerns in the recipients of transcatheter valves. Studies have suggested the role of complex native calcifications of the aortic valve in the occurrence of these complications [7,8]. However, the computed tomography (CT) imaging modalities routinely used in the perioperative work-up for TAVR have proven unable to clearly identify all the anatomical parameters or characteristics predicting the risk of occurrence of device complications [11,12].

**Figure 1:**
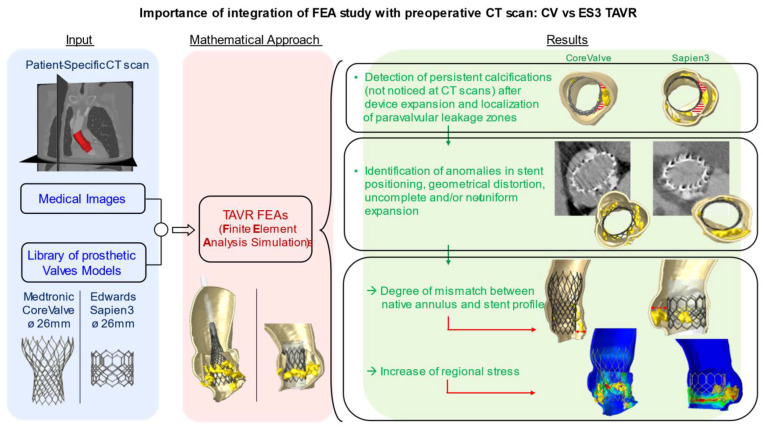
The integration of preoperative medical images with FEA simulation can describe the impact of uncrushed calcifications on TAVR complications. These studies could be useful in predicting potential risk factors.

Finite element analysis (FEA) was shown to provide a more accurate evaluation of several morphological and hemodynamical parameters in the aortic root [13,14,15]. The integration of FEA with the routinely used CT scan could therefore constitute a fruitful approach to investigate factors implied in the development of device complications and to predict their occurrence on the basis of patient-specific imaging. We therefore use post-processing FEA to investigate cases of failed self-expandable and balloon-expandable transcatheter heart valves, with the aim to identify elements or abnormalities potentially responsible for valve failure and to generate potential models to predict the occurrence of complications (Figure 1).

## 2. Methods

Between 2014 to 2018, thirty-nine (6.4%) TAVR recipients were admitted to the Centre Cardiologique du Nord (project identification code (IRB CCN_TAVR_1)) for heart failure related to valve thrombosis, aortic insufficiency, or paravalvular leakage, and underwent a CT scan after TAVR. We selected two patients with device failure to perform post-processing FEA analysis on the basis of the CT scan performed at the moment of rehospitalization. In particular, one patient received a first generation 26-mm CoreValve (Medtronic, Minneapolis, MN, USA) and another received a 26-mm third-generation balloon-expandable Sapien 3 transcatheter heart valve (Edwards Lifesciences Inc., Irvine, CA, USA), both affected by device thrombosis. We compared the information from FEA biomodelling with a similar analysis performed on two control patients who received a routine follow-up CT scan after an uncomplicated TAVR with the same devices.

FEA was therefore used in 4 patients and focused on aortic root anatomical configuration and calcifications distribution with the aim to describe their impact on device implantation and positioning.

### 2.1. Computed Biomodelling Study

Computed biomechanical models were designed to study the first generation 26-mm CoreValve as well as the third-generation 26-mm Edwards Sapien. The CoreValve consists of a self-expanding nitinol frame supporting a trileaflet porcine pericardial valve, while the frame material of the balloon-expandable Edwards Sapien is a cobalt–chromium (CoCr) alloy, with bovine pericardial leaflets. Physical measurements were studied to enable accurate modeling of the valve. For both devices, the process to perform TAVR simulations and analysis included the following: (1) micro-computed tomography (micro-CT) scanning of the valves at 0 mmHg; (2) modeling of the TAVR mesh using 3D geometries of the leaflets and stents; (3) application of material properties of the stents and leaflets followed by systemic pressure loading; and (4) FEA using a finite element solver.

The commercial finite element solver Abaqus 2017 by Dassault Systèmes (Simulia, Providence, RI, USA) was used to create aortic valve finite element models (FEMs) and perform simulations, including stents crimping and prosthesis implantations in the native roots. Preoperative CT images were used to create patient-specific geometrical models of the aortic valve complex. The simulation of TAVR implantation included the following: (1) pre-processing the medical images; (2) identifying appropriate models for analysis; (3) simulation of the procedure on the basis of the acquired data; and (4) post-processing of the simulation results.

### 2.2. Native Aortic Root Model

ITK-SNAP 3.6 software (www.itksnap.org accessed on 20 February 2021) was utilized to identify the main anatomical features of the patient’s aortic root from preoperative DICOM images and generate three-dimensional reconstructions. Following the segmentation procedure, an in-house developed Matlab code was used (v.R2018b, Mathworks Inc., Natick, MA, USA) allowing the generation of a suitable mesh of the aortic wall, assuming a constant thickness of 2.5 mm (for simplicity). Then, Rhinoceros 5.0 commands (McNeel & associates, Seattle, WA, USA), were utilized to construct a definitive 3D CAD (Computer Aided Design) geometry of the aortic root. Volumetric reconstruction of the aortic root was exported to Abaqus for discretization using C3D4 tetrahedral elements.

### 2.3. Calcifications

Calcifications close to the leaflets were included to accurately re-enact TAVR as they may significantly affect the dynamics of stent expansion alongside the technical complexity and efficacy of the procedure [16]. Each calcification was considered as a single entity in the Abaqus model; specifically, a kinematic coupling constraint technique was utilized to rigidly connect the surface nodes of the calcium deposits to the specific reference nodes of the leaflets.

### 2.4. Prosthetic Model

Accurate geometrical models of the 26-mm CoreValve and Edwards Sapien 3 were obtained from high-resolution micro-CT images of the actual devices after their expansion. The CAD models of the stent frames were built using Rhinoceros 5.0 and Matlab. Abaqus/Explicit was used to reproduce the crimping phases of both the devices into their catheters by gradually reducing the initial catheter diameters (42 mm and 27 mm) to the final ones, as follows: 6 mm and 4.7 mm, for CoreValve and Sapien, respectively. Pericardial tissue leaflets were excluded because their effect on mechanical stents performance and interactions with the aortic root wall were negligible.

With the aim to model the mechanics of a self-expandable valve, the gradual removal of the rigid catheter was performed post-compression, with an upwards sliding movement to enable stent reopening. This strategy allowed the CoreValve frame to express its super-elastic tendency, as per the nitinol constitutive laws suggested by Auricchio et al., [17] in both crimping and expansion simulations. In fact, the tensional states of a shape memory alloy (SMA) such as nitinol, achieved during the crimping phase, influence the behavior of the specific material also during its re-expansion process.

On the other hand, starting from the crimped configuration of the Sapien frame, the behavior of the balloon-expandable valve within the patient’s aortic root was reproduced by applying a uniform radial displacement to the nodes of a rigid cylindrical surface, which was assumed to represent the inner expanding balloon.

### 2.5. Material Models

Simplified isotropic St. Venant-Kirchhoff material properties were utilized to model native leaflet tissues, using Young’s modulus *E* of 2 MPa and a Poisson’s ratio *ʋ* of 0.45. The hyperelastic material was described by a six-order reduced polynomial constitutive model, using material parameters proposed by Martin et al. [18] to represent the nearly incompressible behavior of cardiac root tissue. Equal density *ρ* (1.1 × 10^−9^ tonn/mm^3^) was assumed for the aortic wall and leaflets [10], while calcified tissues adopted the following parameters: *E* = 10 MPa, *ʋ* = 0.35, and *ρ* = 2 × 10^−9^ tonn/mm^3^ [19]. Fourteen constants were defined to represent the behavior of the nitinol materials (CoreValve), while the elastoplastic behavior of the Sapien stent was described using a von Mises plasticity model with isotropic hardening. The parameters adopted were set at the following values: *E* = 233 GPa; *ʋ* = 0.35; 414 MPa, 933 MPa, and 44.5% in terms of yield stress, ultimate stress and deformation at break, respectively [20].

### 2.6. Simulation Details

During TAVR simulations, the ratios between kinetic energy and internal energy remained less than 10%, as stent deployment could be considered a quasi-static phenomenon. To capture the in vivo conditions of the aortic roots, preliminary boundary conditions were applied to both extremities (constrained to a normal plan to the axis of the stents), preventing excessive movements, while the nodes at the bottom of the stents were blocked to hinder longitudinal translations of the prosthetic devices during the reopening phase. The time period to simulate the stents implantation inside the patients’ aortic root was set to 0.4 s.

## 3. Results

### 3.1. Prosthetic Stent Deformation

**Figure 2:**
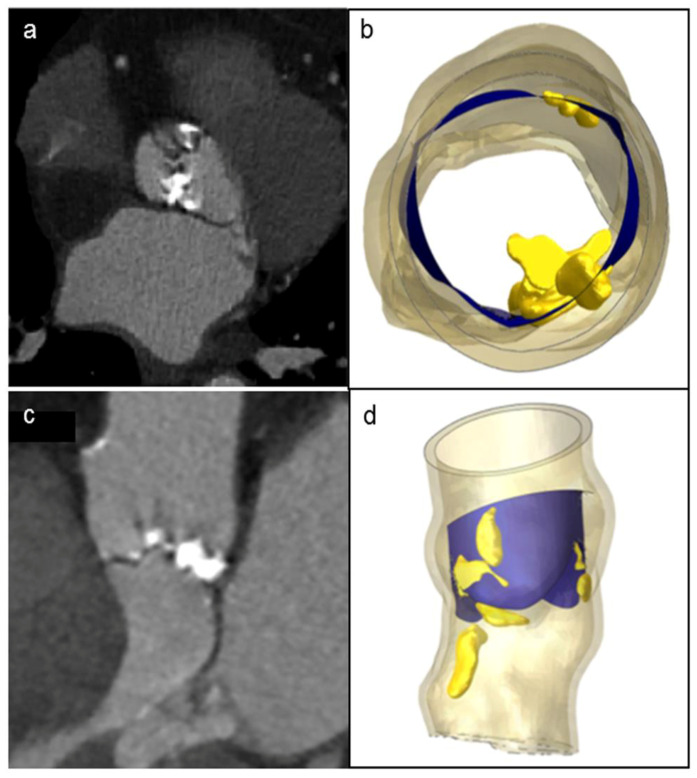
Patient’s preoperative CT scans of severe bulky calcifications attached to aortic valve leaflets and root (**a**,**c**), compared to the model reconstruction (**b**,**d**).

**Figure 3:**
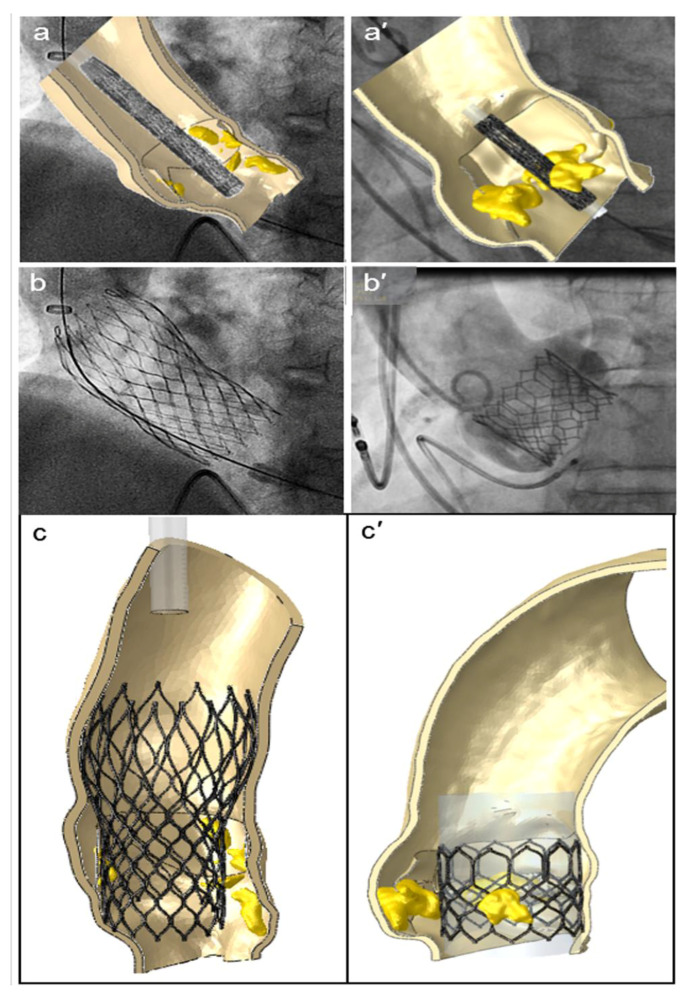
FEA simulations of TAVR in two investigated patients who showed postoperative thrombosis. The positioning (**a**,**a′**) and reopening (**c**,**c′**) of CoreValve and Sapien devices (left and right sides, respectively), compared to the corresponding intraoperative angiographic images (**b**,**b′**).

A patient-specific model of the aortic valve obtained from preoperative CT imaging is shown in Figure 2. FEA simulations of the positioning and deployment of self- and balloon-expandable devices in the two investigated patients who developed complications are depicted in Figure 3a,a′,c,c′, respectively, and compared with corresponding intra-operative images (Figure 3b,b′).

**Figure 4:**
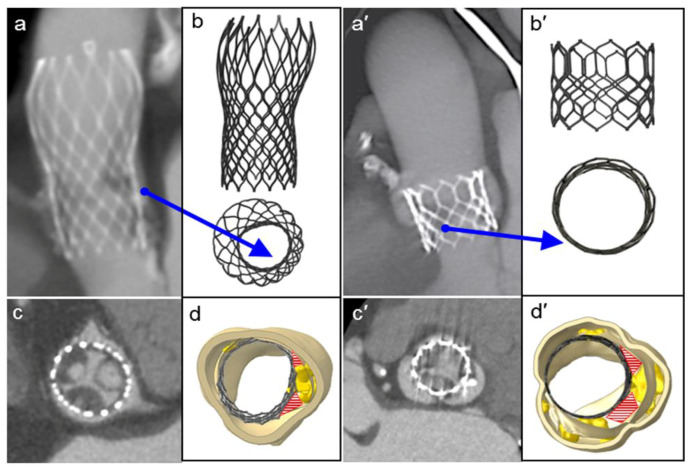
Postoperative medical images (**a**,**c**,**a′**,**c′**) compared to the FEA results of the TAVR simulations (**b**,**d**,**b′**,**d**′, front and top view, respectively) of the CoreValve and Sapien devices. The red areas show paravalvular orifices due to the presence of residual calcifications, and the blue arrows indicate the major distortion of the frames and their incomplete and/or asymmetric expansion.

FEA modeling can evaluate the presence of persistent complex calcifications, which were not identified by the follow-up 3D CT scan. It should be noted that by persistent complex calcifications we mean the calcium agglomerates that are not crushed during the implantation of the device. Importantly, we found that the native morphology of the aortic root, and the quantity and position of the calcium conglomerates, may determine a non-circular stent deformation and its asymmetric expansion with incomplete deployment of both the devices (see blue arrows in Figure 4a,b,a′,b′, for CoreValve and Sapien, respectively). Consequently, prosthetic stent deformation translated into a progressive elliptical shape of the device.

**Figure 5:**
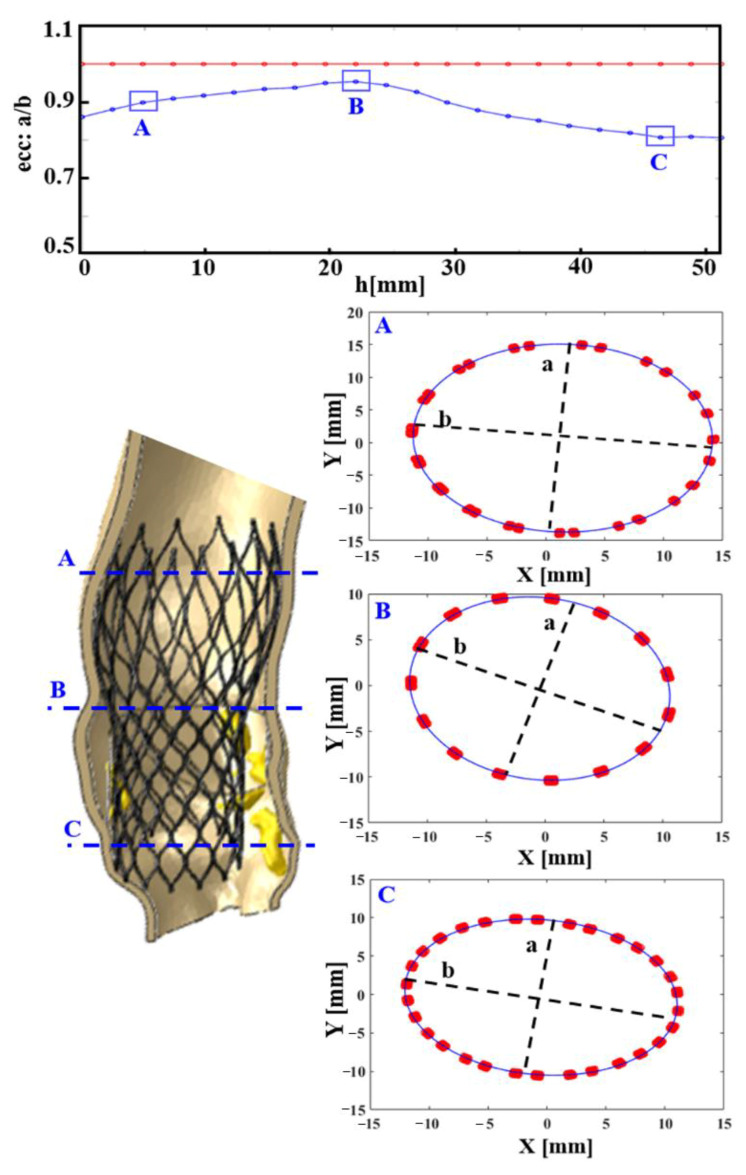
In the re-hospitalized patient: CoreValve deformation measured through its postimplant eccentricity, computed as *a/b*, i.e., (minimum radius)/(maximum radius) of 22 equally spaced cross-sections of the frame. In the first chart from above, the blue line describes the real prosthesis deformation, plotted against its longitudinal height (*h*, growing towards the inferior base of the device), compared to the red one, which represents the perfect circularity (ideal eccentricity *ecc*: *a/b* = 1). Three cross-sections (**A**–**C**) are highlighted. As expected, the most elliptically deformed device portion is the distal one (**C**), at the level of the greatest uncrushed calcifications, (*mm*: millimeter values).

**Figure 6:**
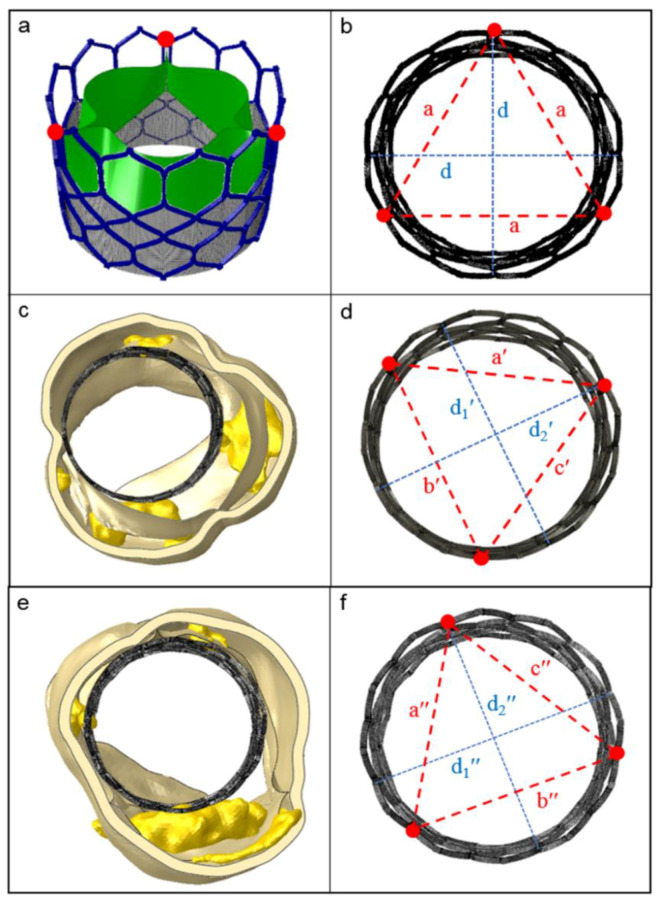
The ideal shape of a Sapien 3 stent frame (upper panels) compared to the real ones after implantation in patients under study. (**a**) Open device configuration before crimping; (**b**) perfect frame circularity, when the two principal axes *d* have the same length, such as the triangle edges *a* (top view); (**c**,**e**) balloon-driven device re-expansion within the investigated patients (thrombotic and control case, respectively); (**d**,**f**) evaluation of the implanted stent deformation, proposed as the measure of the three distances between the points at the commissure windows (red dots). [*a*′ ≠ *b*′ ≠ *c*′ ≠ *a*; *a*″ ≠ *b*″ ≠ *c*″ ≠ *a*; *d*_1_′ and *d*_2_′, *d*_1_″ and *d*_2_″: minor and major axes of the originated ellipses; *d*_1_′ ≠ *d*_2_′ ≠ *d*; *d*_1_″ ≠ *d*_2_″ ≠ *d*].

The eccentricity index (*ecc*) could be calculated as the ratio between the minor and major elliptical axes. The ideal value of this ratio is 1.0, which would imply perfect circularity (see red line in the eccentricity chart, reported in Figure 5). After plotting the cross-sectional device area against its height, we found the maximum values of deformation were >10% for the CoreValve devices in both the analyzed patients (case of postoperative complications in Figure 5, and uncomplicated implantation evaluated as a control in Figure S1, in the Supplementary materials), i.e., *ecc* < 0.9 in correspondence with uncrushed bulky calcifications (see section C; detailed values in Table 1). Figure 6 illustrates the distortions which occurred within the implanted balloon-expandable devices. In a re-hospitalized patient (Figure 5c,d), the elliptical shape was not very pronounced and the stent frame remained almost circular, comparing it to the ideal configuration shown in Figure 5b (*a* = 22.52 mm). Both the minor and major axes lengths *d*_1_′ and *d*_2_′ were very similar, and close to the nominal diameter *d* (*d*_1_′ = 25.68 mm and *d*_2_′ = 25.73 mm vs. *d* = 26 mm), with an index of eccentricity higher than 0.99 (*ecc* = 0.9981). However, the Sapien device expansion was not perfectly symmetrical, as proved from the following differences among the values of the postimplant-derived triangle segments: *a*′ = 22.2 mm, *b′* = 21.42 mm, and *c*′ = 21.79 mm, while the ideal configuration would be *a*′ = *b*′ = *c*′, as proposed by Morganti et al. [10].

Interestingly, when comparing a device that developed thrombosis with that of a patient who underwent uncomplicated TAVR (Figure 5e,f), we noticed that the values defining frame distortion in the successful procedure were closer to the ideal condition, as demonstrated by the higher measured eccentricity (*ecc* = 0.9984). By means of the computational model, it was possible to quantify the following parameters describing stent expansion: *d*_1_″ = 25.72 mm, *d*_2_″ = 25.68 mm, *a*″ = 21.94 mm, *b*″ = 22.39 mm, *c*″ = 21.55 mm, with a consequent overall average distortion of 2.5% (Table 1).

### 3.2. Localization and Evaluation of Paravalvular Leakage

Paravalvular leakage zones were detected in proximity to calcifications which persisted after device expansion (red areas in panels d-d′ in Figure 4d,d′ and Figure S2, in the supplementary materials). These areas led to a significant mismatch between the expanded stent and the internal surface of the aortic root, whereas moderate/severe patient prosthesis mismatch was defined as an indexed EOA ≤ 0.85 cm^2^/m^2^ [21]. At the end of implantation, the basal crown of the stent should completely adhere with the aortic annulus. However, in the analyzed cases, optimal deployment of the stents was hindered by the calcium conglomerates. Considering cross-sections of the examined aortic roots to investigate zones prone to paravalvular leakage, the Sapien prosthetic valve had a localized area of 62.62 mm^2^ associated with PVL (Figure 4d′), while in the CoreValve device (Figure 4d) measured 19.3 mm^2^. The control cases (Figure S2, in the supplementary materials) had smaller PVL areas (13.08 mm^2^ and 4.31 mm^2^, respectively), determining just mild regurgitation (all measurements in Table 1).

### 3.3. Stent–Root Contact Area Measurement and Stress Distribution

**Figure 7:**
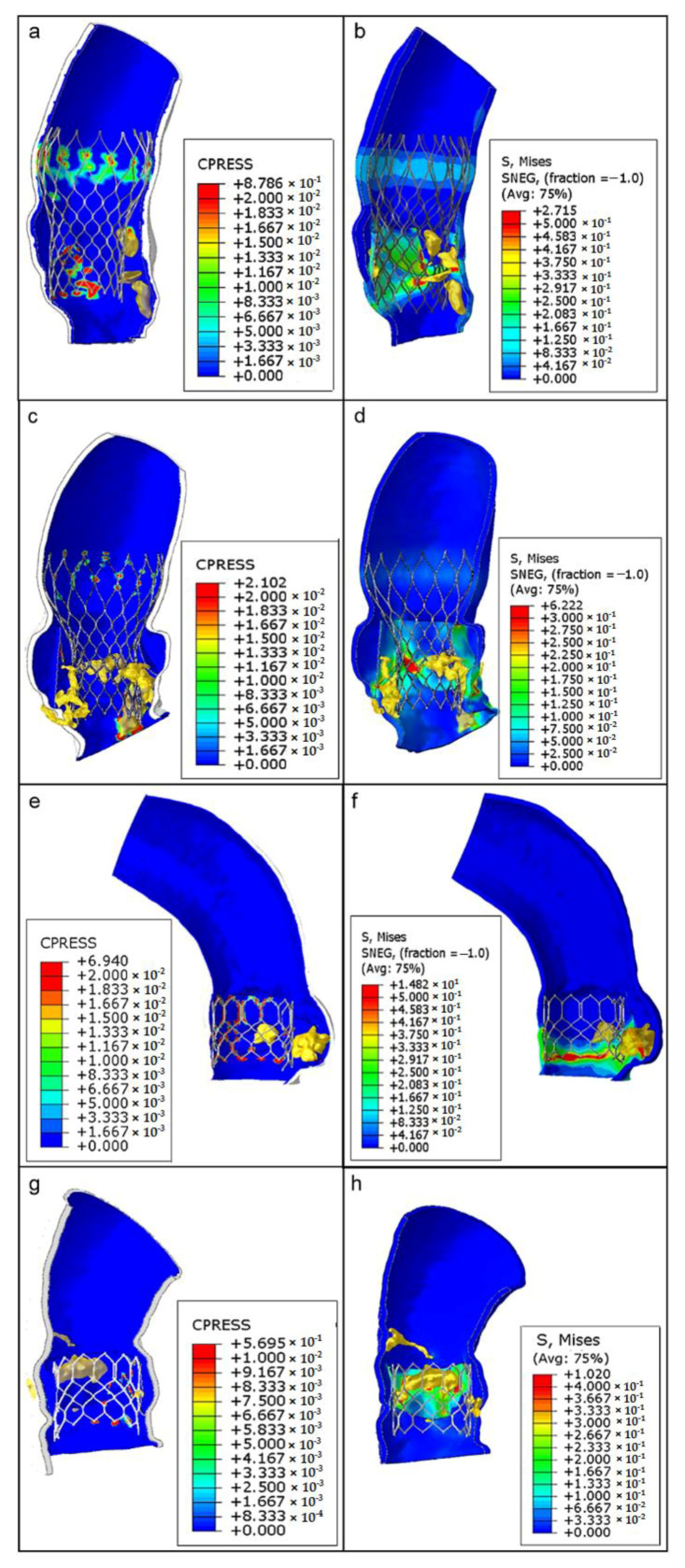
From top to bottom, as follows: CoreValve TAVR with postoperative complications (**a**,**b**), CoreValve implant as a case control (**c**,**d**), Sapien TAVR with consequent thrombosis (**e**,**f**), and Sapien implant without any complications (**g**,**h**). Contact pressure (CPRESS): measurement of the interaction between the expanded device and the inner aortic surface elements (left panels); von Mises average stress distribution (S, MISES): measure of stress induced by the device expansion onto the aortic wall (to the right).

Stent–root contact area (where contact pressure—CPRESS—is induced) at the level of the devices’ anchoring zones provides an indication of the degree of stent apposition (see Figure 7a, the re-hospitalized patient after CoreValve implantation; Figure 7c, the CoreValve control case; Figure 7e,g, Sapien 3 TAVR in patients with and without post-operative complications).

The lower values of pressure (blue areas, around 0 MPa) could suggest a higher risk of stent migration because of the lack of anchorage to the specific aortic root anatomy, while higher pressure values (green–red areas) indicate a good level of apposition. Noteworthy, the areas of lower pressure values and anchorage were located in correspondence with bulky calcific plaques, while the zones characterized by satisfactory apposition are situated particularly opposite to the calcifications (range of values are reported in Table 1).

The analysis of von Mises stresses induced by stent expansion on the inner aortic wall showed non-uniform distributions in each patient; elevated values were concentrated in regions close to solid calcifications and in contact zones between the vessel and the metallic frame of the stents (Figure 7b,d,f,h). As expected, the highest degree of stress (green–red areas) was identified at the zones with the highest contact pressure. These areas may also be at major risk for aortic wall inflammatory changes.

Table 1 summarizes all the numerical results observed in the examined implant simulations for both cases and the uncomplicated controls. Despite the impossibility of statistically assessing these values, it appears that in uncomplicated control subjects the evaluated parameters were on average lower than the corresponding ones in patients who developed thrombosis.

## 4. Discussion

Pathogenesis of RLM, HALT and other device-related complications is multifactorial. The degree of native valve calcifications, stent geometry, and size of the patient’s annulus, alongside the physiological dynamics of blood flow, were demonstrated to affect the performance of TAVR devices [17,22,23]. In first-generation biomechanical models of CoreValve [11] and Sapien XT [10], the presence of isolated bulky annular calcifications was shown to lead to geometric alterations of the aortic annulus post-deployment.

It has been suggested that calcifications persisting after device expansion might be responsible not only for anomalies of stent positioning with subsequent potential PVL, but also for dynamic alterations hampering leaflet movement and inducing RLM and HALT [24]. However, routine CT scan is not able to detect and integrate these morphological and hemodynamic data, thus necessitating the development of more comprehensive imaging techniques.

In this study we combined CT imaging and post-processing of finite element analysis simulation, to evaluate a subgroup of failed TAVR in which massive thrombosis developed after the index procedure. These data were compared to TAVR without further complications. To our knowledge, this is the first study conducting FEA modelling of both CoreValve and Sapien 3 transcatheter valves.

Our analysis revealed the presence of calcifications that were not detected on the post-operative CT scan after the TAVR procedure. It is important to underline that unlike the standard aortic valve replacement, which allows the complete removal of calcifications, in the percutaneous procedure the calcifications are pressed against the aortic wall. The postoperative CT scan fails to show these calcifications [11,12]. Prosthetic stent deformation of both self-expandable and balloon-expandable devices was noted as a consequence of these calcifications, which caused a high degree of mismatch between the native aortic root wall and the stent profile. The non-uniform expansion, due to persistent bulky calcifications, led to different degrees of eccentricity, which were more significant for the CoreValve devices. This finding could be attributed to the nitinol stent of this valve which is a more deformable material than the cobalt–chromium alloy contained in Sapien 3. This led to incomplete deployment of the metallic frame at almost all levels, resulting in an elliptical configuration of the device. Sites of PVL were located in correspondence with these regions of geometrical alterations and mainly situated between the non-expanded stent and the internal aortic root.

Importantly, FEA established a link between the presence of these calcifications and the aforementioned elliptical deformations of the stent, as shown by the higher values of von Mises average stress in these regions. It is possible to hypothesize that this geometrical alteration might hamper leaflet mobility and trigger hemodynamical abnormalities and flow turbulence, generating a thrombotic nidus for subsequent HALT. This event has been shown to be prodromic to the occurrence of increased transvalvular gradients and early structural valve deterioration, and to be associated with increased risk of ischemic accidents [7,8,25]_._

Unfortunately, in the current study no dynamic fluid modelling and integration with complex fluid–structure interaction simulations were possible to confirm hemorheological abnormalities or stasis within the aortic sinuses. However, taking into consideration the morpho-functional findings of our FEA simulations, and the recent data from the Galileo trial on the efficacy of rivaroxaban in HALT [26], it is reasonable to associate thrombus formation with prosthetic frame distortion. Despite its speculative nature, this study might provide a link between the presence of residual native calcifications, subsequent device distortion, leaflet hypomobility, and the development of valve thrombosis.

The use of balloon- and self-expandable catheter-based aortic valves merits a careful preoperative evaluation with advanced and functional imaging to define the optimal positioning strategy [16,17,27]. The use of FEA as an integration with routine imaging for TAVR could provide valuable information to this regard, and reveal links between adverse anatomical characteristics and the subsequent development of device complications. Despite preliminary and based on a retrospective analysis, the results of this study permitted to generate hypotheses on the association between post-procedural persistent calcification and the risk of TAVR complications as thrombosis and paravalvular leaks. If confirmed by larger prospective studies, the predictive capacity of FEA modeling could be exploited as a valid adjunct to CT scans in the clinical practice, during TAVR work-up. Furthermore, FEA analysis could inspire the design of novel tissue-engineered heart valves or aortic root substitutes.

## 5. Study Limitations

There are several limitations of the biomechanical analysis in our study. Firstly, a combined analysis of CT scans and FEA with biomechanical modelling was possible only for four patients (two patients having undergone TAVR with the CoreValve device and two patients with the Edwards Sapien device) because of the high complexity of the model. This inevitably hindered the possibility of statistical calculations. However, given the accuracy of our geometrical models, the mathematical reiterations inherent in their construction, and in the execution of the analysis, we can reliably assume the reproducibility of the results. Also, other studies performed using a similar approach included a similar number of cases [13,14,15].

Although in the current model crimping and ballooning processes were considered, the risk of early structural valve deterioration due to the microscopic leaflet damage could also play a role, as demonstrated in ex vivo studies [28,29]. In fact, the thinner, newer pericardial valves might be even more sensible to crimping [25,27], especially considering the progressive reduction in sheath sizes used for device delivery (up to 14 F size). Clearly, further studies on leaflet stress and damage could be of help to evaluate the risks of accelerated structural valve degeneration [30].

Furthermore, a study of the complex fluid–structure interaction simulations was not performed, thus limiting comparative analysis based on dynamic fluid models. Although these were beyond the field of application of this study, it is well known that they play a role in understanding the development of thrombosis.

Finally, the present study does not include simulations of surgical aortic valve replacement of the same size, which may be used to obtain exact quantitative comparisons with pericardial valves.

## 6. Future Perspectives

FEA was able to provide a detailed picture of the morphological characteristics of the aortic root and a high-fidelity simulation of the changes occurring after device implantation. This permitted the hypothesis of the potential pathogenic links between the anatomical features of the native root, the characteristics of the devices implanted and their functional consequences. In particular, root calcifications persisting after device implantation might be responsible for stent deformation, potentially favoring the onset of device complications. The currently used CT imaging is unable to provide such details and to suggest the potential development of complications because it lacks the possibility to simulate device implantation in the specific patient’s root anatomy. If confirmed by further studies, the use of FEA as an adjunct to routine preoperative imaging would be a valuable tool to assist patient selection and procedure planning as well as help in the detection and prevention of major TAVR complications. In fact, being built on the basis of preoperative imaging, FEA can generate patient-specific simulations and assist the development of more tailored approaches in TAVR. Additionally, the detailed information acquired on aortic root and valve anatomy could inform in the future the design of tissue-engineered valve substitutes.

## Figures and Tables

**Table 1:** Postprocessing of FEA results.

TAVR Procedure	ecc [−] (Avg Distortion)	PVL Area [mm^2^]	S, Mises [MPa]		CPRESS [MPa]	
			Min	Max	Min	Max (Contact Area)
26 mm CV_th	0.80–0.95 (≈12.5%)	19.34	0.0–0.17	0.21–2.71	0.0	0.88 (886.4 mm^2^)
26 mm CV_ctrl	0.75–0.99 (≈13.4%)	4.31	0.0–0.1	0.12–6.22	0.0	2.1 (168.9 mm^2^)
26 mm ES_th	>0.99 (3.2%)	62.62	0.0–0.17	0.21–14.82	0.0	6.94 (232.4 mm^2^)
26 mm ES_ctrl	>0.99 (2.5%)	13.08	0.0–0.13	0.17–1.02	0.0	0.57 (108.2 mm^2^)

A comparison among different values of parameters measured through biomechanical analysis in the investigated patients. The patient without postoperative complications (*_ctrl*) presented lower PVL area (mild regurgitation), as desirable. The Sapien 3 prosthetic valve with postimplant thrombosis (*ES_th*) showed the worst results in terms of PVL, values of stress and pressure. The red values are associated with increased risk of complications. (*Avg*: average; *CV_th*, *CV_ctrl*, *ES_th*, *ES_ctrl*: patients who underwent TAVR by CoreValve (*CV*) and Sapien3 (*ES*) devices and presented postoperative complications (*th*) or not (*ctrl*)).

## Supplementary Materials

The following are available online at https://www.mdpi.com/article/10.3390/bioengineering8050052/s1, Figure S1. CoreValve postimplant eccentricity for control case: in the first chart from above, the blue line describes the real prosthesis deformation, plotted against its longitudinal height (h, growing towards the inferior base of the device), compared to the red one, which represents the perfect circularity (ideal eccentricity ecc: [minimum radius]/[maximum radius]: a/b = 1). 20 equally-spaced cross-sections of the frame were evaluated, and three of them are highlighted (A-C): as expected, the most elliptically deformed device portion is the distal one (C), at the level of the greatest uncrushed calcifications, as shown by the deviation of blue curve from the red one. On the other hand, the middle portion of this implanted CoreValve is the most circular (note the proximity of the blue point B –corresponding to central slice– to the circularity). [mm: millimeters values]. Figure S2. FEA simulation of TAVR in patients without postoperative complications: comparison between CT scans and FEA implant simulations (a and b, a′ and b′) for CoreValve and Sapien3 devices, respectively; (c and d, c′ and d′) top view of the aortic root at the end of the procedure: red areas indicate PVL caused by calcifications, clear also from medical images (see blue arrows). Video 1 Patient-specific CoreValve-26mm implantation and clinical outcome. Video 2 Patient-specific Sapien3-26mm implantation and clinical outcome.

## Abbreviations

TAVR	Transcatheter aortic valve replacement
CT	Computerized tomography
THVT	Transcatheter heart valve thrombosis
PVL	Paravalvular leakage
FEA	Finite element analysis
DAPT	Dual antiplatelet therapy medication
RCT	Randomized clinical trial
HALT	Hypo-attenuated leaflet thickening
