# Supplementary material for: CoreValve vs. Sapien 3 Transcatheter Aortic Valve Replacement: A Finite Element Analysis Study
Source: Bioengineering (Basel). 2021 Apr 27;8(5):52. doi: 10.3390/bioengineering8050052 (PMC8146716; doi:10.3390/bioengineering8050052)
Supplement: Supplementary file 1 [file bioengineering-08-00052-s001.zip › bioengineering-1136440-supplementary.pdf]

Supplementary Material

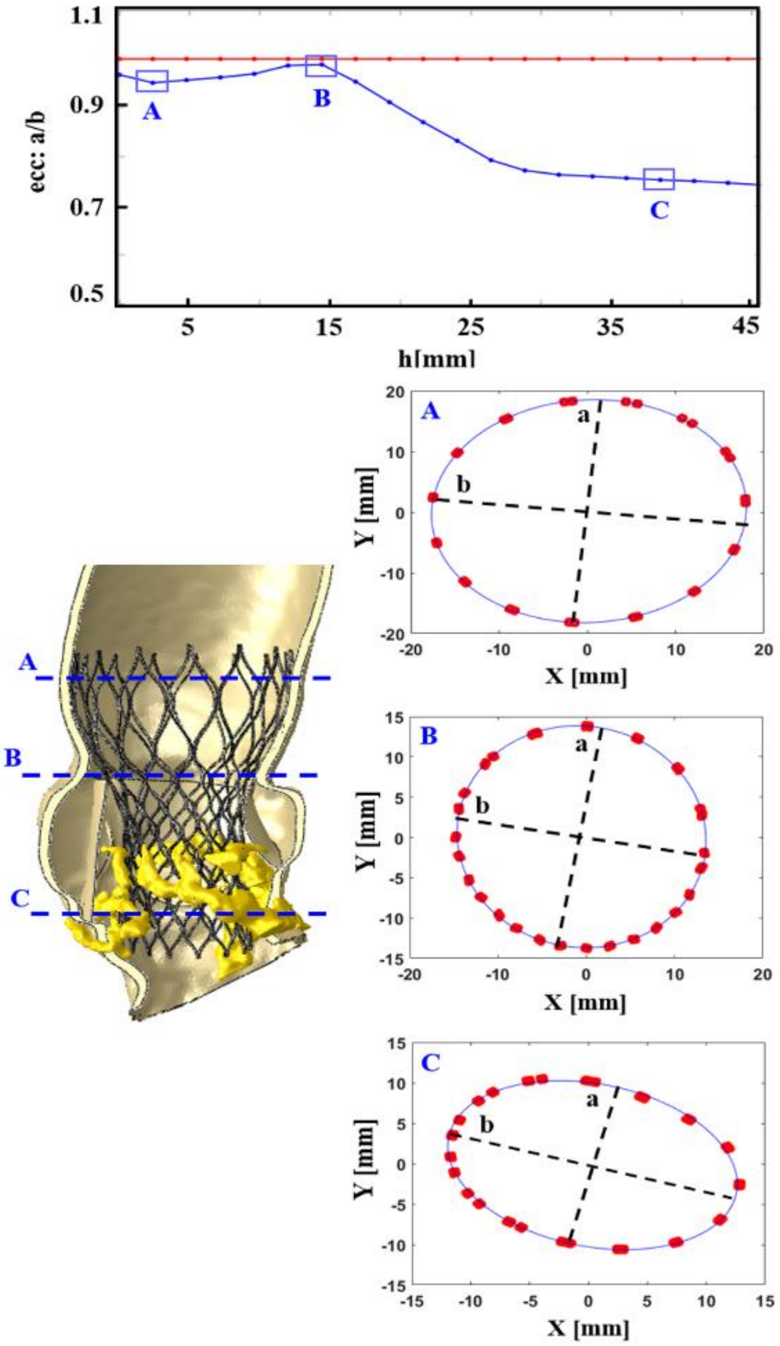

Figure S1. CoreValve postimplant eccentricity for control case: in the first chart from above, the blue line describes the real prosthesis deformation, plotted against its longitudinal height (h, growing towards the inferior base of the device), compared to the red one, which represents the perfect circularity (ideal eccentricity ecc: [minimum radius]/[maximum radius]: a/b = 1). 20 equally-spaced cross-sections of the frame were evaluated, and three of them are highlighted (A-C): as expected, the most elliptically deformed device portion is the distal one (C), at the level of the greatest uncrushed calcifications, as shown by the deviation of blue curve from the red one. On the other hand, the middle portion of this implanted CoreValve is the most circular (note the proximity of the blue point B –corresponding to central slice– to the circularity). [mm: millimeters values].

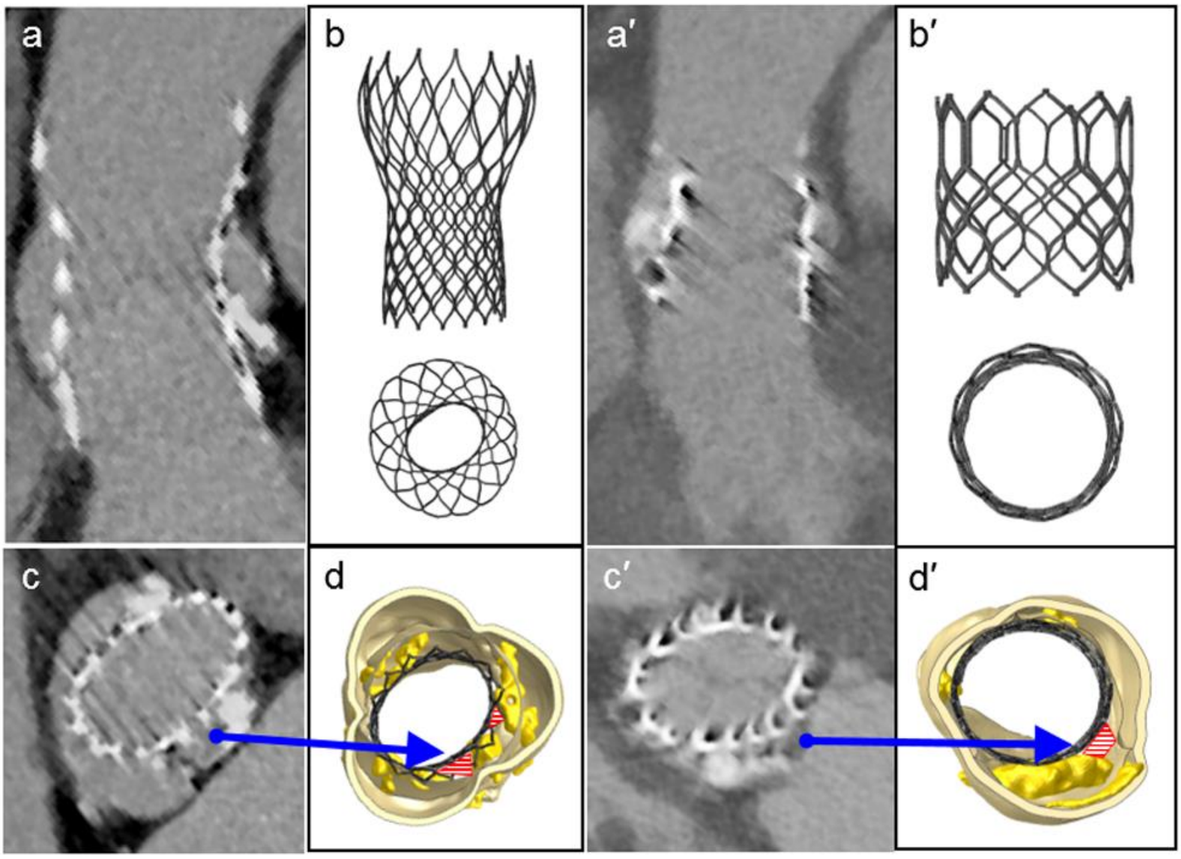

Figure S2. FEA simulation of TAVR in patients without postoperative complications: comparison between CT scans and FEA implant simulations (a and b, a′ and b′) for CoreValve and Sapien3 devices, respectively; (c and d, c′ and d′) top view of the aortic root at the end of the procedure: red areas indicate PVL caused by calcifications, clear also from medical images (see blue arrows).

Video 1 Patient-specific CoreValve-26mm implantation and clinical outcome.

TAVR performed in an 83-year-old woman with prohibitive surgical risk (preoperative image in the first frame). Success of the implanted CoreValve in clinical practice and simulation of the same procedure in the evaluated patient (video time step t = 41 seconds), which exhibits the same outcome. Postoperative CT images at one year (t = 55-57 sec): persistent calcification and obstruction of TAVR (red arrows). Last frame clearly shows thrombosis at the level of posterior and anterior-right leaflet, extended to the outer surface of the CoreValve.

Video 2 Patient-specific Sapien3-26mm implantation and clinical outcome.

TAVR performed in an 83-year-old man. The first three frames in preoperative phase (t = 0-5 sec) display calcifications (see red arrows). Following frames show the correctly executed implantation of the device both in clinical imaging (balloon inflation, subsequent deflation, and blood perfusion) and FEA simulation (from t = 24 sec). Postoperative images (from t = 34 sec) highlight the presence of bulky calcifications (particularly, see red arrows in the last frame) causing thrombus.
